# A blood–brain barrier overview on structure, function, impairment, and biomarkers of integrity

**DOI:** 10.1186/s12987-020-00230-3

**Published:** 2020-11-18

**Authors:** Hossam Kadry, Behnam Noorani, Luca Cucullo

**Affiliations:** 1 Department of Pharmaceutical Sciences, Jerry H. Hodge School of Pharmacy, Texas Tech University Health Sciences Center, 1300 S. Coulter Street, Amarillo, TX 79106 USA; 2 Dept. of Foundational Medical Studies, Oakland University William Beaumont School of Medicine, Office 415, Rochester, MI 48309 USA

**Keywords:** Blood–brain barrier, Tight junctions, Transcytosis, Disruption, Permeability, Markers, CNS, Neuroinflammation, Degenerative, TEER, Integrity

## Abstract

The blood–brain barrier is playing a critical role in controlling the influx and efflux of biological substances essential for the brain’s metabolic activity as well as neuronal function. Thus, the functional and structural integrity of the BBB is pivotal to maintain the homeostasis of the brain microenvironment. The different cells and structures contributing to developing this barrier are summarized along with the different functions that BBB plays at the brain–blood interface. We also explained the role of shear stress in maintaining BBB integrity. Furthermore, we elaborated on the clinical aspects that correlate between BBB disruption and different neurological and pathological conditions. Finally, we discussed several biomarkers that can help to assess the BBB permeability and integrity in-vitro or in-vivo and briefly explain their advantages and disadvantages.

## Background

**Fig. 1:**
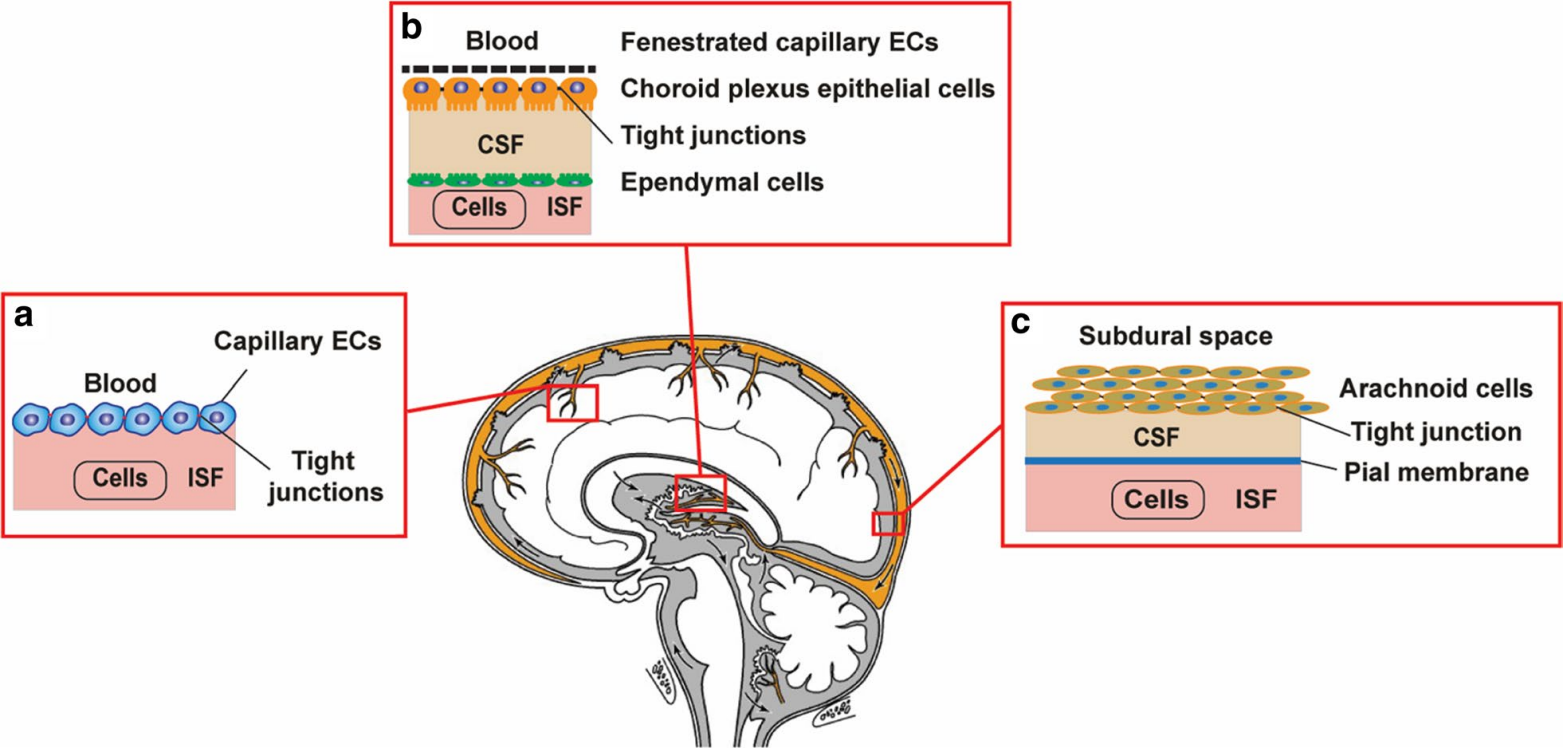
Biological barriers are protecting the brain. **a** Blood–brain barrier; **b** blood–CSF barrier; **c** the arachnoid barrier

A highly controlled microenvironment is required to promote the normal functioning of the central nervous system (CNS). The existence of a biological barrier at the blood to brain interface effectively separating the brain from the rest of the body was established after the finding of Paul Ehrlich when he noticed that a peripherally infused dye did not stain the brain tissue. His finding was further supported by later observation from his associate Goldmann as he applied the same dye to the cerebrospinal fluid. It did stain only the brain tissue without extravasating in the periphery [1]. These biological barriers are established by different cells at three key interfaces: the blood–brain barrier (BBB), blood–CSF barrier (BCB), and the arachnoid barrier [2] (see Fig. 1).

The BBB is formed by microvascular endothelial cells lining the cerebral capillaries penetrating the brain and spinal cord of most mammals and other organisms with a well-developed CNS [3]. It is considered the largest interface for blood–brain exchange as the combined surface area per average adult is assumed to fall between 12 and 18 m^2^ based on an average microvessels surface area of 150 and 200 cm^2^ per gram tissue [4]. The BBB is playing a critical role in protecting the brain parenchyma from blood-borne agents and providing a significant obstacle to the entry of drugs and other exogenous compounds into the central nervous system.

The second barrier is the blood–cerebrospinal fluid barrier (BCSFB), which is formed by the epithelial cells of the choroid plexus. The CSF secretion into the ventricular brain system is controlled by the choroid plexus epithelial cells [5]. In contrast, the interstitial fluid (ISF), which is consisting of the rest of the brain extracellular fluid, is derived at least partially by secretion across the capillary endothelium of the BBB [6–9]. ISF communicates freely with CSF over several locations, and its contribution to CSF is estimated between 10 and 60% [2].

Underlying the dura mater, we find the avascular arachnoid epithelium, which is considered the third barrier. The dura mater covers the CNS and completes the seal between the extracellular fluids of the central nervous system and the rest of the body [10]. Its contribution to the exchange between blood and brain is insignificant due to its avascular nature and the relatively small surface area compared to other barriers [11].

In this review, we will focus more on BBB as it is the main barrier contributing to CNS protection and maintaining the brain homeostasis. Unlike other vascular endothelial cells lining peripheral blood vessels, brain microvascular endothelial cells present distinctive morphological, structural, and functional characteristics that set them apart from other vascular endothelia. These include the following: (1) the expression of tight junctions (TJs), sealing the paracellular pathways between adjacent endothelial cells, thus preventing the unregulated passage of polar (water-soluble) molecules between the blood and the brain; (2) the absence of fenestrations; (3) the lack of pinocytic activity and the expression of active transport mechanisms to regulate the passage of essential molecule (including nutrients and essential amino acids) while blocking the passage of potentially undesired substance (both endogenous and xenobiotics) [12]. The transport barrier comprehends various efflux transporters, such as P-glycoprotein (P-gp) [13], breast cancer resistance protein (BCRP) [14], organic anion transporting polypeptide (OATP) [15]. These efflux systems may also share overlapping substrate affinities (such as P-gp and BCRP—[16]) actively pump compounds (including xenobiotics) out of the endothelial cells back into the blood circulation [17], resulting in reduced CNS exposure. Lastly, the presence of drug-metabolizing enzymes within the brain endothelial cells, such as CYP 450 enzymes (CYP1B1 and CYP2U1, CYP-3AF), results in the formation of the metabolic barrier [18–21]. CYP mRNAs have been found in several areas of the human brain which plays a role in neurodegenerative disease by metabolizing drugs and fatty acids [20]. For instance, CYP2U1 metabolizes fatty acids like arachidonic CYP1B1 has a role in the metabolism of endogenous compounds in the CNS [19]. CYP3A4 also showed to be functionally expressed at drug resistance of epileptic BBB and oxidizes a large group of xenobiotics, including antiepileptic drugs [22].

**Fig. 2:**
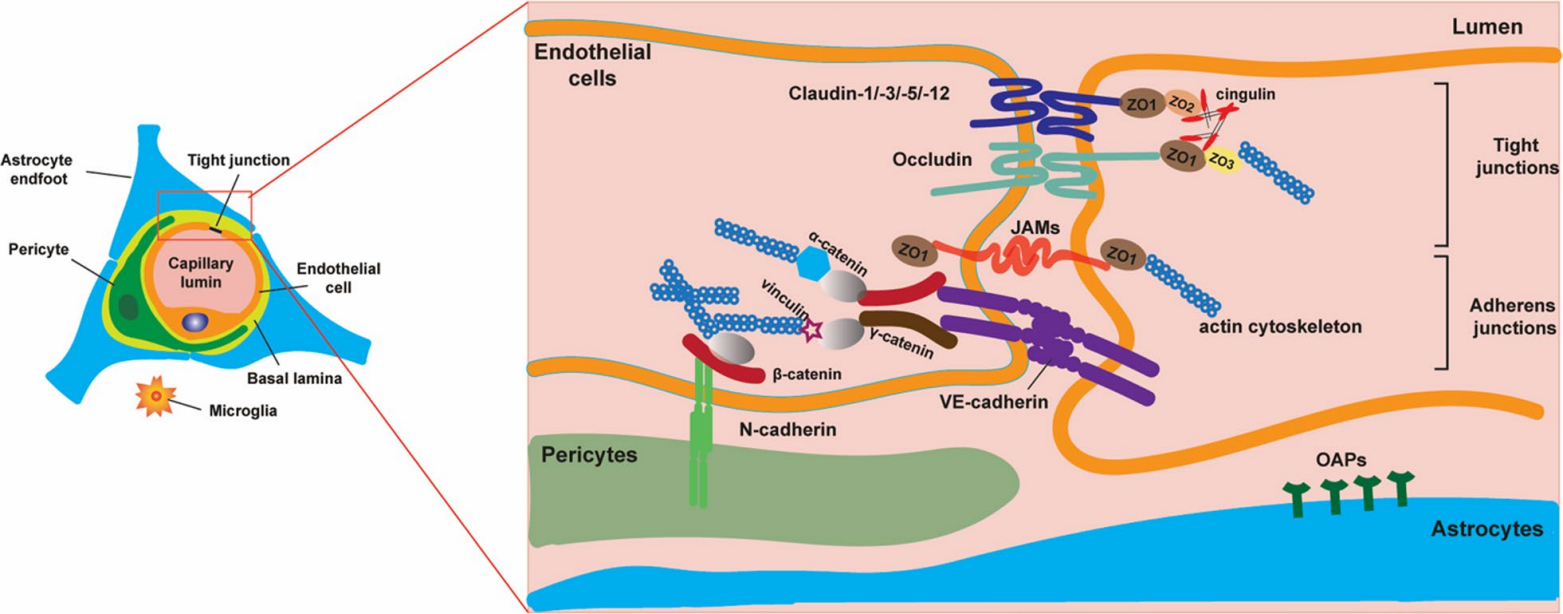
Cells association at- and molecular organization of the neurovascular unit (NVU)

These integrated barrier systems, while protecting the brain from potentially harmful compounds, are also the major obstacle for delivering drugs into the CNS [23]. Overall, the BBB function as a dynamic interface regulating the brain homeostasis and protecting the CNS, which can respond to different physiological and pathological conditions [10]. The Induction and maintenance of the barrier function fall primarily on the interaction between the microvascular endothelium, the astrocytic foot processes (which invest close to 99% of the abluminal surface area of the brain capillary), and pericytes [24] (see also Fig. 2).

In addition to microvascular endothelial cells, the capillary basement membrane (BM), astrocytes, pericytes (PCs) embedded within the BM, microglial and neuronal cells complete the structure of what we now refer to as the neurovascular unit (NVU) [25]. Below we provide a brief description of these cells and their role in maintaining the integrity of the BBB.

## Different cells of the NVU

### Endothelial cells

The BBB endothelial cells (ECs) in the mature mammalian brain are characterized by different features, which make them phenotypically different from other ECs located at different parts of the body. ECs are characterized by a flattened appearance, the expression of inter-endothelial tight junctions, the presence of very few caveolae at the luminal surface, and a high number of mitochondria [26] when compared to ECs from other vascular districts. During embryonic angiogenesis, the differentiation of the endothelium into a functioning barrier layer begins and is maintained during adulthood by a close inductive association with other cell types within the NVU, as we mentioned early [2].

The paracellular flux of hydrophilic molecules across the BBB endothelium is hindered by the tight junctions sealing the paracellular pathways between adjacent endothelial cells. The TJs also provide a fence around the cell, separating its luminal portion from the basolateral region [1]. Across the endothelium, there is rapid free diffusion of oxygen from the blood to the brain and carbon dioxide diffusion in the opposite direction, which is essential for normal brain metabolism and regulation of pH in the brain ISF, neurons, and other NVU cells. Besides, small lipophilic molecules, with a molecular weight (MW) < 400 Da forming < 8 hydrogen bonds, can cross the BBB [27]. Glucose, amino acids, and other nutrients enter the brain via carrier-mediated transporters. In contrast, the uptake of larger molecules such as insulin, leptin, and iron transferrin are facilitated via receptor-mediated endocytosis [28, 29].

### Pericytes

Pericytes are essential constituents of the brain capillary with different frequencies in different vascular beds. They are most abundant in the CNS, particularly in the retina [1]. They share a basement membrane with endothelial cells and form direct synaptic-like peg-socket focal contacts with endothelium through N-cadherin and connexins [30] (see also Fig. 2). They can be visualized immunohistochemically by using antibodies against smooth muscle actin, desmin, platelet-derived growth factor b-receptor (PDGFR-b), aminopeptidase N, or the regulator of G-protein signalling-5 (RGS5) [31–33].

Their close association with ECs allows the exchange of ions, metabolites, second messengers, and ribonucleic acids between the two cell types [30]. Pericytes also play essential roles in maintaining BBB integrity**,** aiding in angiogenesis, and microvascular stability [30, 34]. Pericytes, which also feature contractile characteristics similar to smooth muscle cells, can regulate (to some extent) the capillary diameter and the cerebral blood flow (CBF) [35, 36]. Furthermore, pericytes may display phagocyting functions helping with the removal of toxic metabolites [37]. They have also been reported to have multipotent stem cell capabilities [38]. Studies have shown that PCs express receptors for vascular mediators, such as catecholamines [39], angiotensin I [40], vasoactive intestinal peptides [41], endothelin-1 [42], and vasopressin [43]. These data strongly suggest that PCs play an essential role in cerebral autoregulation.

Besides, in vitro studies [44] have shown that ECs associated with PCs are more resistant to apoptosis than isolated endothelial cells, further supporting the role of PCs in supporting the structural integrity and genesis of the BBB. The loss of PCs and the formation of microaneurysm in PDGF-B deficient mice suggest that PCs play an essential role in the maintenance of vascular wall stability [45]. These data are noteworthy from a clinical standpoint since the degeneration and injury of PCs have been reported in many neurological diseases, including Alzheimer’s disease (AD) [46–49], mild dementia [50], amyotrophic lateral sclerosis (ALS) [34], and stroke [35].

### Astrocytes

ACs represent the most abundant cells in CNS and are involved in different physiological and biochemical tasks. These latter include (1) compartmentalization of the neural parenchyma; (2) maintenance of the ionic homeostasis of the extracellular space; (3) pH regulation; (4) neurotransmitter uptake and processing by providing energy-rich substrates to the neurons; (5) mediation of signals from neurons to the vasculature [51].

In particular, ACs are believed to play a decisive role in maintaining the barrier function of the brain microcapillary ECs and in controlling the CBF [2]. The astrocytic foot processes establish the direct interface between the vascular compartment and the neuroglial in the NVU. Recent literature stressed the role of astrocytic endfeet as key checkpoints of brain metabolism [52]. At the contact interface between astroglial endfeet and the superficial or perivascular basal lamina, there is a great density of intramembranous organic anion transporters (OAPs). OAPs’ density decreases where the glial cell membrane loses contact with the basal lamina [53]. This polar heterogeneity of astrocytic membrane domains represents one of the most impressive morphological features of mammalian and avian ACs, which seems to be correlated with the BBB maturation during development [1].

The contribution of different NVU cells in developing and maintaining the BBB have supported by several grafting and cell culture studies. When avascular tissue brain graft from a 3-day old quail was transplanted into the coelomic cavity of chick embryos, the endothelial cells vascularizing the graft formed a competent BBB [54]. In contrast, transplanting avascular embryonic quail coelomic graft into embryonic chick brain resulted in leaky capillaries and venules within the mesenchymal tissue of the graft. When cultured astrocytes were introduced into areas with normal leaky vessels, they induced tightening of the endothelium [55]. Another study showed that the optimal generation of BBB necessitates direct contact between endothelial cells and astrocytes [56].

The induction of BBB characteristics in ECs may be attributed not only to the NVU cells, but their cell-derived soluble factors as human or bovine endothelial cell monolayers showed a higher transendothelial resistance when being cultured in astrocyte-conditioned media [57]. Astrocytes participate in the dynamic regulations of the neural system and play a vital role in CNS inflammation in neurodegenerative diseases [58].

## Tight junctions

Tight and adherens junction (AJs) constitutes the junction complex of the BBB. The TJs are present at the sites of fusion involving the outer surface of the plasma membrane of adjacent endothelial cells or the same cell (see Fig. 2). Adherens junctions are composed of a cadherin–catenin complex and its associated proteins. The TJs consists of three integral membrane proteins, namely, claudin, occludin, and junction adhesion molecules (JAMS), and several cytoplasmic accessory proteins including Zonula occludens-1, -2, -3 (ZO-1, ZO-2, ZO-3), cingulin, and others (see Fig. 2). The cytoplasmic proteins link membrane proteins to actin, which is the primary cytoskeleton protein for the maintenance of structural and functional integrity of the endothelium.

After early detection of the cytoplasmic accessory proteins, intramembrane junctional proteins were found to be directly contributing to the restricted interendothelial permeability. Occludin, a protein with four transmembrane domains, was the first discovered protein, which seemed to play a less important role in permeability restriction as occludin-deficient mouse mutant is viable and has intact biological barriers. The discovery of claudins, another four transmembrane domains protein with no homology to occludin, was of fundamental importance for TJs research. Also, JAMs and the endothelial selective adhesion molecule (ESAM), which are members of the immunoglobulin superfamily, are considered TJs components, which probably fulfill regulatory functions of the barrier [59].

Tight junction formation appears to be a very early feature of BBB development, and a barrier to the free movement of proteins and macromolecules is formed at the primary stages of brain development [2].

### Claudins

Claudins-1 and -2 are 22 kDa phosphoproteins with four transmembrane domains. These claudins were identified as an integral component of TJs strands [60]. Claudins are the major components of TJs, where they bind homotypically to claudins on adjacent endothelial cells to form the primary seal of the TJ [61]. Claudins are linked to cytoplasmic proteins, including ZO-1, ZO-2, and ZO-3, through their carboxy-terminal [61]. In the brain, claudins-1 and -5, besides occludin, are the major components of endothelial TJs forming the BBB [62, 63]. In vivo and in vitro studies showed the loss of claudin-1 from cerebral vessels and ECs under pathologic conditions such as cancer, stroke, inflammation [62, 64, 65]. Also, the selective loss of claudin-3 or occludin was observed in experimental allergic encephalomyelitis (EAE) and glioblastoma multiform (GBM) resulting in a loss in BBB integrity along with some functional barrier loss [66]. The disappearance of claudin-5 from the tight junctional complexes can result in a compromised BBB as mice genetically altered to lack claudin-5 have shown severely compromised and leaky BBB and die shortly after birth [67], although their death is probably not solely related to the BBB defects [2]. Double knockdown of claudin-5 with occludin also increased the permeation of tracers between 3 and 10 kDa [68]. Furthermore, knock-down of claudin-5 was found to change the mRNA expression of other TJ proteins such as claudin-1 [69]. Previous studies have been shown that claudin-5, not only regulates paracellular ionic selectivity but also played a role in the regulation of the endothelial permeability in several pathological processes containing inflammation, trauma, toxic damage, and tumor cell motility [70, 71]. For instance, Persidsky et al. [72] showed that the downregulation of TJ proteins, claudin-5, and occludin, resulting in decreased barrier tightness and enhanced monocyte migration across the BBB during human immunodeficiency virus-1 (HIV-1) encephalitis.

Recently, the claudin-11 gene and protein were identified in brain endothelial cells and capillaries. It was originally recognized as oligodendrocyte-specific protein [73] and later named to the claudin protein family [69]. The partial overlap of Claudin-11 with claudin-5 was observed in the capillaries of the human brain section. It has been shown that claudin-11 plays a role in paracellular tightness by homophilic oligomerization [69]. Moreover, Uchida et al. [74] showed that claudin -11 was found to be significantly decreased in the brain and spinal cords capillaries of patients with MS and experimental autoimmune encephalomyelitis (EAE) mice.

### Occludin

Occludin, a 65-kDa phosphoprotein, was first discovered by immunogold freeze-fracture microscopy in chickens [75] and then in mammals [76]. Also, the expression of occludin has been reported in rodents [77] and adult human brain [78] but not in the human newborn and fetal brain. Occludin is significantly larger than claudins and shows no amino acid sequence similarity with them. It has four transmembrane domains-as claudins, a long COOH-terminal cytoplasmic domain, and a short NH_2_-terminal cytoplasmic domain. The two extracellular loops of occludin and claudin originating from neighboring cells form the paracellular barrier of TJs while the cytoplasmic domain of occludin is directly associated with ZO proteins. Occludin is highly expressed in brain ECs compared to nonneural tissues and seems to be a regulatory protein that has a crucial role in paracellular permeability [77].

Occludins and claudins assemble into heteropolymers to form intramembranous strands, which have been proposed to contain fluctuating channels allowing the selective diffusion of ions and hydrophilic molecules [79]. Breakdown of the BBB in the tissue surrounding brain tumors occurs with concomitant loss of a 55-kDa occludin expression [78]. Claudins and occludins form the extracellular component of TJs and are both essential for the formation of the BBB [80].

### Junctional adhesion molecules

Junctional adhesion molecules are recently identified 40 kDa proteins that belong to the immunoglobulin superfamily [81]. They have a single transmembrane domain, and their extracellular portion presents two immunoglobulins like loops that are formed by disulfide bonds. Studies in rodent brain sections showed that JAM-1 and JAM-3 are expressed in the brain blood vessels but not JAM-2 [82]. Studies also showed their role in cell-to-cell adhesion and monocyte transmigration across BBB [82, 83]. However, more investigations are required to unveil its function in the BBB.

### Cytoplasmic accessory proteins

Zonula occludens proteins (ZO-1, ZO-2, and ZO-3), cingulin, and several other proteins are essential for TJs functioning as they form the cytoplasmic bridge connecting the TJs to the cell cytoskeleton. ZO-1 (220 kDa), ZO-2 (160 kDa), and ZO-3 (130 kDa) have sequence similarity with each other and belong to the family of proteins known as membrane-associated guanylate kinase-like protein (MAGUKs). They contain three PDZ domains (PDZ1, PDZ2, and PDZ3), one SH3 domain, and one guanyl kinase-like (GUK) domain. These domains function as protein-binding molecules and thus play a role in organizing proteins at the plasma membrane. The PDZ1 domain of ZO-1, ZO-2, and ZO-3 has been reported to bind directly to the COOH-terminal of claudins [84]. For Occludin, the GUK domain is the site of interaction with ZO-1 [85]. Recently, studies show the ability of JAM to bind directly to ZO-1 and other PDZ-containing proteins [86]. Importantly, actin, the primary cytoskeleton protein, binds to the COOH-terminal of ZO-1 and ZO-2, which stabilizes transmembrane elements and provides structural support to the endothelial cells [87].

## Adherens junctions

Cadherin is the main component of these junctional membrane proteins, which joins the actin cytoskeleton through intermediary proteins, catenins, to form adhesive contacts between cells. AJs assemble via homophilic interactions between the extracellular domains of calcium-dependent cadherin on the surface of adjacent cells. The submembrane proteins β- or γ-catenin connects the cytoplasmic domains of cadherins to the actin cytoskeleton via α-catenin (see Fig. 2). AJs components, including cadherin, alpha-actinin, and vinculin (α-catenin analog), have been demonstrated in intact microvessels of the BBB in rats. TJs and AJs components are known to interact, particularly ZO-1 and catenins, and influence TJs assembly [79].

The intracellular scaffold proteins ZO-1, ZO-2, and ZO-3, which link the junctional molecules claudin and occludin via cingulin to intracellular actin and the cytoskeleton appear to play a crucial role in the efficiency of the TJs [52, 59, 88]. Studies show that changes in calcium concentration both intracellular and extracellular can have a great impact on tight junction assembly and efficiency as a barrier and can alter the electrical resistance across the cell layer [10, 89]. As mentioned early, soluble factors released by many of the cell types associated with brain microvessels (including microglia and astrocytes) as well as nerve terminals adjacent to the endothelial extracellular matrix/basal lamina (such as vasoactive agents and cytokines) can modify tight junction assembly and barrier permeability [10, 90].

## Hemodynamic modulatory functions in BBB physiology: role of shear stress

Mammalian endothelial cells are known to undergo dynamic changes under different stimuli. Cellular, molecular, and physical stimuli are the essential players that help ECs to acquire specialized functions. For most cell types, chemical signaling seems to play a pivotal role in cell physiology. Physical stimulation, such as shear stress, is one of the most important but underestimated physiological stimuli, which contributes to vascular ECs differentiation and maturation besides other cellular and molecular signaling. Akin to responses to inflammatory cytokines, shear stress has been shown to cause dramatic changes in ECs morphology [91], gene expression [92], and function [93]. Shear stress showed to induce the production of reactive oxygen species (ROS) and nitric oxide (NO). NO is known to cause vasodilation, autocrine signaling, and also increase the production of free radicals. A study showed that ECs might scavenge free radicals by increasing levels of GAPDH and other intracellular enzymes [94]. Another study showed that NO might have a protective role in BBB during reperfusion after the transient loss of flow in a condition mimicking ischemic stroke [95].

Shear stress was shown to induce a significant upregulation of tight and adherens junction proteins and genes in human brain microvascular endothelial cells (HBMECs). The study reported a 5.91- and 2.13-fold increase in claudin-5 and cadherin-5 gene expression, respectively. HBMECs showed higher maturation and differentiation under shear stress, as indicated by the increase in the trans-endothelial electrical resistance (TEER) from 100 to 700 Ω cm^2^. Also, SS induced the endothelial expression of different drug transporters and metabolic properties that allow the BBB to protect the CNS from harmful substances [96]. In contrast, a study on iPSCs-HBMECs d didn't show any significant changes in tight junctions, TEER, or cell morphology. These discrepancies highlight the importance of further studies to optimize culture models based on non-primary BBB ECs to better reflect the physiological responses of the BBB in vivo [97].

Akin to cytokines effect, shear stress has been shown to play a critical role in maintaining blood vessels’ homeostasis and a protective role. A study in HBMECs showed that shear stress was a much more potent stimulus for thrombomodulin (TM) release, yielding media TM levels of 1000 pg/10^5^ cells when compared to 175 and 210 pg/10^5^ cells for, tumor necrosis factor-α (TNF-α) and interleukin-6 (IL-6), respectively. Thrombomodulin is an integral membrane receptor constitutively expressed on the luminal surface of vascular ECs and an essential determinant of blood vessel homeostasis. The study also showed that shear-conditioned media was able to completely block thrombin-induced permeabilization of HBMECs, which confirm the protective role of shear stress [98]. Overall, BBB studies must consider the effect of shear stress as it is playing an essential role in BBB maturation and homeostasis. In this respect, our group has summarized the advances in in-vitro BBB models [99, 100].

## Physiological functions of the BBB at the blood–brain interface

### Maintain ionic homeostasis and brain nutrition

The BBB provides a controlled microenvironment via a combination of specific ion channels and transporters, which keep the ionic composition optimal for neural and synaptic signaling functions. For instance, the levels of potassium in CSF and ISF is maintained at ~ 2.5–2.9 mM. In comparison, plasma concentration is approximately 4.5 mM, despite fluctuations that can occur in potassium plasma levels following exercise or a meal, imposed experimentally, or resulting from pathology [101, 102]. Other ions such as calcium and magnesium and pH are also actively regulated at the BBB and BCSFB [103, 104]. Calcium and potassium homeostasis controls neuronal excitability but is also essential for the transmigration of macrophage across the BBB [105]. Furthermore, Ca^2+^ is involved in the modulation of BBB integrity and endothelial morphology [106].

Specific ion channels and transporters at BBB provides the optimal preservation environment for synaptic and neural activity. As an example, the abluminal sodium pump (the Na^+^, K^+^-ATPase) maintains a high concentration of Na^+^ and low levels of K^+^ in brain ISF via transporting Na + into the brain and K + out of the brain. Or the luminal Na^+^, K^+^, Cl^−^ cotransporter facilitate the transfer of Na^+^, K^+^, 2Cl^−^ from blood to the endothelium. Calcium transporters (the Na^+^–Ca^2+^ exchanger) and voltage-gated K^+^ channel also regulate the ion transport across the BBB [107, 108].

For essential water-soluble nutrients and metabolites required by nervous tissue, the BBB allows for low passive permeability. In contrast, for other nutrients that cannot pass, there are specific transport systems expressed in the BBB to ensure an adequate supply of these substances. The selective and region-specific (luminal and abluminal surfaces of the ECs) expression of these transporters confers the normal polarity of the BBB endothelium [10, 52]. The differentiation of the endothelium into a barrier layer begins during embryonic angiogenesis and in the adult is primarily maintained by a close inductive association with several cell types, especially the endfeet of astrocytic glial cells.

### Regulate levels of neurotransmitters

The central and peripheral nervous systems share many of the same neurotransmitters, so the BBB helps to keep the central and peripheral transmitter pools separate, minimizing ‘crosstalk’ and protecting the brain from unexpected changes in their plasma levels. For example, blood plasma contains high levels of the neuroexcitatory amino acid glutamate, which fluctuate significantly after the ingestion of food. High levels of glutamate in the brain ISF will have harmful effects on neuronal tissues. An example is a case of glutamate secretion from hypoxic neurons during ischemic stroke, which results in considerable and permanent neurotoxic/neuroexcitatory damage to neural tissue [10, 109].

The transfer of neurotransmitters from the brain to blood primarily dependent on Na^+^-coupled and Na^+^-independent amino acid transporters. The BBB limits the influx of some amino acids including the neurotransmitters glutamate and glycine, while it effluxes many other essential amino acids. Hladky and Barrand reviewed comprehensively the transport of amino acids across the BBB with different transport systems based on the type of amino acids [108, 110].

### Limit plasma macromolecules leak into the brain

The production of CSF from plasma, under normal condition, passed through an efficient filtration process in the choroid plexus to remove unneeded plasma proteins. This process helps in controlling the protein content of CSF and results in minimal quantities of proteins in CSF compared to the plasma protein levels [2].

Under physiologic conditions, the BBB prevents many macromolecules from entering the brain through normal paracellular or diffusion routes. The leakage of these large molecular weight serum proteins into the brain across a damaged BBB can have severe pathological consequences. For example, the leakage of plasma proteins such as albumin, prothrombin, and plasminogen has a detrimental effect on nervous tissue, causing cellular activation, which can lead to apoptosis [111, 112]. There is a wide distribution of different activators for these proteins within the CNS. These include factor Xa, which converts prothrombin to thrombin, or tissue plasminogen activator, which converts plasminogen to plasmin. The resulting proteins, thrombin or plasmin, can bind to their receptors in brain tissue and initiate cascades resulting in seizures, glial activation, glial cell division and scarring, and cell death [113]. Thus, the BBB works as a “gatekeeper,” allowing the entry of only the beneficial materials.

### Protect the brain against neurotoxins

Many potential neurotoxins are circulating in our blood, including those from endogenous sources such as metabolites or proteins, or exogenous ones such as xenobiotics ingested in the diet or otherwise acquired from the environment. The BBB function is to regulate the entry of different circulating substances based on CNS needs. The transport barrier represented by multiple ABC energy-dependent efflux transporters (ATP-binding cassette transporters) occupies the BBB luminal surface. It actively pumps many of these agents out of the brain [2]. The adult CNS has a limited regenerative capacity if damaged, and fully differentiated neurons have a minimal ability to divide and replace themselves under normal circumstances. There is a continuous steady rate of neuronal cell death from birth throughout life in the healthy human brain, with relatively low levels of neurogenesis [114]. That is why any factor promoting an acceleration of the natural rate of cell death (e.g., increased access of neurotoxins into the brain) would become prematurely debilitating.

## Transport across the BBB

### Passive diffusion

In general, a wide range of lipid-soluble molecules can diffuse passively through the BBB and enter the brain [115]. The lipid solubility of a drug is determined by calculating its logD (octanol/water) partition coefficient at pH 7.4. There is a general correlation between the rate at which a solute enters the CNS and its lipid solubility [116]. In contrast with logP, which only considers the partitioning of unionized species, logD includes unionized and ionized species present in solution [116]. Molecular weight is another crucial factor in determining the free diffusion of small molecules across the BBB. Once the MW is > 400 Da, the BBB permeability of the drug does not increase in proportion to lipid solubility [117]. Lipid soluble small molecular compounds are believed to cross the BBB via transitory formation of pores within the phospholipid bilayer that is created as the free fatty acyl side-chains kink in the process of normal molecular motion within the phospholipid bilayer [118, 119]. As the pores are of finite size, they restrict the movement of small molecules that have a spherical volume larger than the pore volume. An increase of the surface area of a drug from 52 A^2^ (e.g., a drug with a MW of 200 Da) to 105 A^2^ (e.g., a drug with a MW of 450 Da) dramatically decrease its BBB permeation [117].

**Fig. 3:**
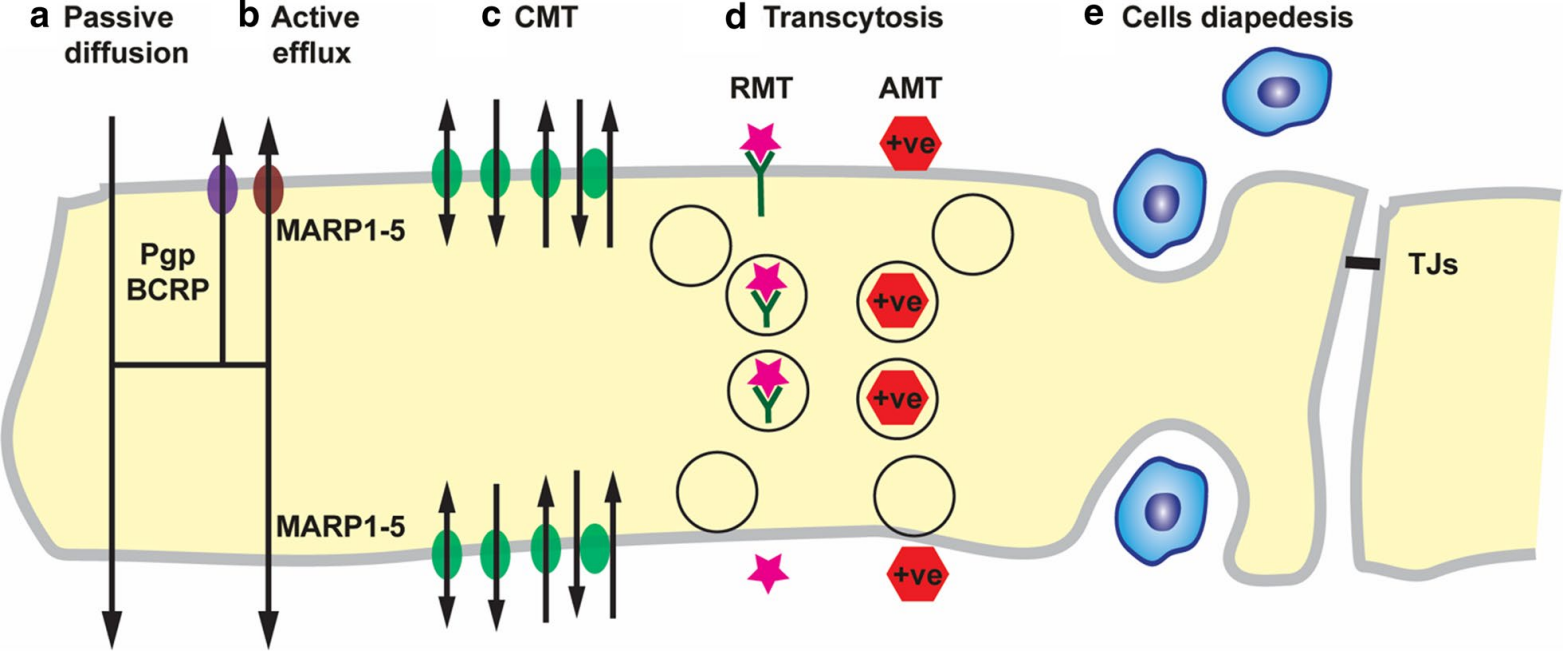
Different methods of transport across the BBB. *CMT* carrier mediated transport, *RMT* receptor-mediated transport, *AMT* adsorptive mediated transport

Also, a high polar surface area (PSA) greater than 80 Å^2^, and a tendency to form more than six hydrogen bonds are considered a limiting factor for the entry of compounds into the CNS. As a general rule, the BBB permeability of a drug decreases 1 log order in magnitude for each pair of H-bonds added to the molecule in the form of polar functional groups [120]. The number of H-bonds that drug forms with water can be calculated by inspecting the chemical structure [121]. Once the number of H-bonds is greater than eight, it is unlikely that the drug crosses the BBB via lipid-mediated free diffusion in therapeutically relevant amounts. Studies showed that the negative effect of H bonds on drug permeability might be attributed to the significant increase in the free energy required for moving the drug from an aqueous phase into the lipid of the cell membrane [116, 122]. The presence of rotatable bonds in the molecule and a high affinity of binding to plasma proteins with a low off-rate can also significantly reduce CNS penetration. It is no assured that drug meeting all the above criteria will be able to cross the BBB and give its action as it may increase the likelihood of becoming a substrate for active efflux transporter [123, 124]. A common misconception is that small molecules readily cross the BBB. However, > 98% of all small molecules do not cross the BBB either. There are > 7000 drugs in the Comprehensive Medicinal Chemistry (CMC) database, and only 5% of these drugs treat the CNS [125]. Figure 3 represents the different transport mechanisms across the BBB.

### Active efflux

Several ATP-binding cassette (ABC) proteins are expressed on the luminal, blood-facing endothelial plasma membrane of the BBB. They are ATP-driven efflux pumps for xenobiotics and endogenous metabolites, which limit the permeability of multiple toxins, including therapeutic agents [126]. The pharmacoresistant characteristics of the CNS are attributed to their high expression. Decreased expression and/or functional activity of ABC BBB transporters were reported in different pathological conditions such as in patients with Alzheimer’s disease (AD) and Parkinson’s disease (PD) [25]. In an animal model of AD, ABC transporters are affected, which leads to the accumulation of amyloid β-peptide (Aβ) in the brain [127].

The primary efflux transporters at the BBB are the P-glycoprotein (Pgp—Multidrug Resistance Protein ABCB1), the Multidrug Resistance-associated Proteins (MRPs, ABCC1, 2, 4, 5 and possibly 3 and 6), and Breast Cancer Resistance Protein (BRCP, ABCG2). Pgp and BCRP are highly expressed in the luminal membrane of the BBB and are responsible for transporting substrate from endothelium to blood; recent studies report some cooperativity of action [127] and substrate overlap [16]. On the other hand, MRP isoforms appear to be expressed in either the luminal or the abluminal membrane [128]. As they favor water-soluble conjugates as substrates, a bi-directional efflux from the endothelium may be predicted, as conjugation by drug transforming enzymes will render them less cytotoxic.

While the brain ECs are considered the primary barrier interface, the transport activity of both pericytes [129] and perivascular astrocytic endfeet [52] may contribute to the barrier function, and may act as a ‘second line of defense’ if the primary barrier is breached or dysfunctional.

### Carrier-mediated transport (CMT)

The BBB isolates the brain and limits the diffusion of many essential polar nutrients, including glucose and amino acids, which are essential for metabolism. Therefore, other routes for the essential nutrients to reach the brain are necessary. CMTs are encoded genes within the Solute Carrier (SLC) Transporter Gene Family. This includes more than 300 transporter genes encoding membrane-bound proteins that facilitate the transport of a wide array of substrates across biological membranes [130]. The SLC transporters facilitate the transcellular movement of a variety of molecules. These include amino acids, carbohydrates, monocarboxylic acids, fatty acids, hormones, nucleotides, organic anions, amines, choline, and vitamins.

Preferential distribution of these transporters over both sides of BBB confer the characteristic polarized behavior of BBB as some of these transport proteins are expressed on either the luminal or abluminal membrane only. In contrast, others are inserted into both membranes of the ECs [128, 131–133]. The orientation of these transporters may, therefore, result in preferential transport of substrates into or across the endothelial cell, and the direction of the transport may be from blood to brain or vice versa.

The tight junctions preserve the polarity of the BBB as they segregate transport proteins and lipid rafts to either the luminal or abluminal membrane domain and prevent their free movement from one side of the endothelium to the other [2].

### Receptor-mediated transport (RMT)

The presence of peptide bonds limits the larger peptides and proteins from using the amino acid CMT systems to cross the BBB [134]. However, specific neuroactive peptides [135], regulatory proteins, hormones, and growth factors get the use of RMT systems to cross the BBB [136]. These Large molecular weight solutes can enter the CNS intact via endocytotic mechanisms in a process named transcytosis. Although most large blood-borne molecules are physically prevented from entering the brain by the presence of the BBB and TJs, specific and some non-specific transcytotic mechanisms exist to transport a variety of large molecules and complexes across the BBB.

There are two types of vesicular transport systems; one is based on receptor-mediated transcytosis (RMT) and the other on adsorptive-mediated transcytosis (AMT). In RMT, macromolecular binds to ligands specific receptors on the cell surface, which triggers an endocytotic event. Both receptors and their bound ligand cluster together, and a caveola are formed, which pinches off into a vesicle. Both ligand and receptors are internalized into the ECs and directed across the cytoplasm to be exocytosed at the opposite side of the cell [2]. Finally, the ligand and receptor dissociate during cellular transit or the exocytotic event (see also Fig. 3). While in AMT, positively charged large molecules interact with specific cell surface binding sites that induce endocytosis and subsequent transcytosis [137].

The lysosomal compartment within the cell needs to be avoided to achieve transcytosis of an intact protein or peptide. This is achieved by directing the primary sorting endosome and its contents away from this degradative lysosomal compartment. Lysosome escaping mechanisms appear to be a specific feature of the BBB endothelium not observed in many peripheral ECs [4]. Another specific feature of BBB endothelium is the presence of relatively few endocytotic vesicles in the cytoplasm of these cells compared to other endothelia, as observed in most electron microscopic studies [138]. However, there is a weak correlation between the protein permeability of a microvessel and the observable endocytotic activity [139]. Brain capillary endothelial cells are very thin, with the luminal and abluminal membranes being only separated by ~ 500 nm (5000 Å) or less. Caveolae are 50–80 nm in diameter, and thus the events of transcytosis may be difficult to capture within the cell using conventional electron microscopical techniques [2].

The RMT receptors may mediate functionally different processes, including (1) transcytosis of the ligand from blood to brain as in the case of insulin and transferrin [140, 141]; (2) reverse transcytosis from the brain to blood such as the transcytosis of IgG via Fc receptor (FcR) [142]; or (3) only endocytosis into the brain capillary endothelium without net transport across the endothelial cells. This latter includes scavenger proteins that facilitate the uptake of acetyl Low-Density Lipoprotein (LDL) from the blood into the BBB endothelium [143] and might be the reason why proteins targeting LDL related receptor type 1 are taken up by the brain at reduced rates compared with proteins that target other transcytosis receptor systems. Studies show that the brain uptake of melanotransferrin (p97) or angiopep-2, ligands that target lipoprotein receptor-related protein 1 (LRP1), is only 0.2% to 0.3% of injected dose (ID)/g in the mouse [144]. This is tenfold lower than the brain uptake of a monoclonal antibody (MAb) that targets the BBB transferrin receptor in the mouse [145].

The fascinating ability of RMT to transport intact large molecules peptides and proteins excite researchers to get the advantage of these receptors in drug delivery in what is called Trojan horses. Molecular Trojan horses are genetically engineered proteins that cross the blood–brain barrier (BBB) via endogenous RMT processes. They allow for the non-invasive delivery of large molecule therapeutics to the human brain [146]. Certain peptidomimetic monoclonal antibodies undergo RMT across the BBB in vivo. The receptor-specific mAb binds to the endogenous BBB peptide receptor at a site that is spatially distant from the endogenous ligand-binding site and carried across the BBB on the endogenous peptide RMT system. One of the most potent BBB molecular Trojan horses is a mAb for the human insulin receptor (HIR), which is active at both the BBB of humans and Old-World primates such as Rhesus monkeys [147]. Besides, molecular Trojan horses targeting the transferrin receptors (TRF) in rats and mice, which showed excellent efficiency. However, these receptor-specific mAbs are species-specific, which suggests that molecular Trojan horses that are used in preclinical research may require further development and optimization to be successfully used as human therapeutics. More efforts need to be directed toward the development of BBB drug targeting technology using molecular Trojan horses as it seems to be a promising technique [148].

### Major facilitator superfamily

Essential omega-3 fatty acids, such as docosahexaenoic acid (DHA), are transported into the brain by the endothelial facilitator superfamily domain-containing protein 2a (MFSD2a) [149]. This family of transporters may have dual function as recent studies showed its importance in maintaining BBB integrity as mice lacking MFSD2a show brain DHA deficits and compromised BBB [150, 151].

### Immune cell movement across the BBB

The CNS is considered as an immune-privileged site as a result of the low infiltration of neutrophils into the brain compared to other tissues and the strictly regulated immune cell-BBB interaction. Under normal physiological conditions, mononuclear cells enter the brain during embryonic development and become resident immunologically competent microglia [152]. They penetrate by process of diapedesis directly through the cytoplasm of the endothelial cells and not via a paracellular route involving re-arrangement and opening of the tight junctional complexes as had been previously suggested [153]. However, in inflammatory pathological conditions, the TJs between endothelial cells may be disrupted. This is the result of cytokines and other pro-inflammatory agents. Furthermore, mononuclear leukocytes, monocytes, and macrophages can enter the CNS via transcellular and paracellular routes and play roles complementary to those of the resident microglia [154, 155]. In some cases, these immune cells may transform into a microglial phenotype [2].

The transmission of immune cells across the BBB is a dynamic process carried out through a sequence of steps including tethering, rolling, crawling, arrest, and diapedesis across the ECs [156]. Multiple studies showed that during inflammation, ECs upregulate expression of vascular cell adhesion molecule 1 (VCAM-1) and intercellular adhesion molecule 1 (ICAM1) which resulted in arresting of CD4+ T cells onto the inflamed CNS vessels or primary brain EC monolayers through the interaction between αLβ2 [lymphocyte function-associated antigen 1 (LFA-1)], and α4β1 [very late antigen 4 (VLA4)] integrin of CD4+ T cells with ICAM-1 and VCAM-1 [156]. Moreover, it has been shown that the polarization and crawling of CD4+ T cells onto the inflamed vessel happened through the interactions of LFA-1-ICAM-1 [156].

Other studies indicated that the transmigration of CD4+ and CD8+ T cells across the CNS autoimmunity could be controlled through additional cell adhesion molecules such as melanoma cell adhesion molecule (MCAM) and activated leukocyte cell adhesion molecule (ALCAM) [157, 158].

Furthermore, it was demonstrated that tight junction and adhesion molecules including claudin-5, VE-Cadherin, JAM, PECAM-1, and CD99 played important roles in the paracellular migration of cell across the BBB [159]. Winger et al. [160] indicated that blocking CD99 in ameliorated EAE mice, diminished the accumulation of CNS inflammatory infiltrates, including dendritic cells, B-cells, and CD4+ and CD8+ T-cells. Administration of anti-CD99 was effective at the initiation of the disease symptoms and also blocked relapse when administered therapeutically after the recurrence of the symptoms [160].

## BBB disruption in different pathological conditions

BBB dysfunction is reported in many CNS pathological conditions including multiple sclerosis [161]; hypoxic and ischemic insult [162]; Parkinson’s and Alzheimer’s disease [163]; epilepsy [164]; brain tumors [165]; glaucoma [166], and lysosomal storage diseases [167]. The observed barrier dysfunction can range from mild and transient changes in BBB permeability, resulting from tight junction opening, to chronic barrier breakdown, and changes in transport systems and enzymes can also occur. This process can also be associated with the degradation of the basement membrane [168]. Microglial activation and infiltration of different plasma components and immune cells into the brain parenchyma result in disturbance of CNS homeostasis and variable damage to the surrounding brain. In most cases, it is not possible to determine whether barrier compromise is causal in disease onset or a result of neurological disease progression. Still, barrier disturbance can often be seen to contribute to and exacerbate developing pathology [169].

As discussed earlier, under normal conditions, the BBB is relatively impermeable. In pathologic conditions, several vasoactive agents, cytokines, and chemical mediators are released that increase BBB permeability. Several in vitro and in vivo studies showed the opening effect on BBB of several mediators include glutamate, aspartate, taurine, ATP, endothelin-1, NO, TNF-α, and macrophage-inflammatory protein 2 (MIP2) which are produced by astrocytes [132, 170, 171]. Other humoral agents reported to increase BBB permeability are bradykinin, 5HT, histamine, thrombin, UTP, UMP, substance P, quinolinic acid, platelet-activating factor, and free radicals [132, 172, 173]. They are variable in the source as some of these agents are released by endothelium and have an autocrine effect on endothelium itself. For example, endothelin (ET-1) acts on Endothelin A (ETA) receptors in ECs. In physiologic conditions, chemical mediators released by nerve terminals of neurons associated with blood vessels such as histamine, substance P, and glutamate, influence and regulate BBB permeability. Table 1 summarizes different pathological conditions that are affecting BBB integrity.

**Table 1:** Different CNS pathological conditions involving BBB disruption

CNS pathology	BBB dysfunction
Stroke	Astrocytes secrete transforming growth factor-β (TGFβ), which downregulates brain capillary endothelial Expression of fibrinolytic enzyme tissue Plasminogen activator (tPA) and anticoagulant thrombomodulin (TM) Proteolysis of vascular basement membrane/matrix Induction of aquaporin 4 (AQP4) mRNA and protein at BBB disruption
Trauma	Bradykinin (an inflammatory mediator) stimulates the production and the release of interleukin-6 (IL-6) from astrocytes, leading to the opening of the BBB
Infectious or inflammatory processes	e.g., bacterial infections, meningitis, encephalitis, and sepsis The bacterial protein lipopolysaccharide (LPS) affects the permeability of BBB tight junctions. This is mediated by the production of free radicals, IL-6, and IL-1 β Interferon-β prevents BBB disruption Alterations in P-glycoprotein expression and activity in the BBB Increased pinocytosis in brain microvessel endothelium and swelling of astrocytes end-feet
Multiple sclerosis	Breakdown of the BBB Tight junction abnormalities Downregulation of laminin in the basement membrane Selective loss of claudin3 in experimental autoimmune encephalomyelitis
HIV	BBB tight junction disruption Cytokines secretion by activated macrophages and astrocytes, e.g., TNF-α, NO, platelet-activating factor, and quinolinic acid
Alzheimer’s disease	Decreased glucose transport, downregulation of glucose transporter GLUT1, altered agrin levels, upregulation of AQP4 expression Accumulation of amyloid-β, a key neuropathological feature of Alzheimer’s disease, by decreased levels of P-glycoprotein transporter expression Altered cellular relations at the BBB, and changes in the basal lamina and amyloid-β clearance
Parkinson’s disease	Dysfunction of the BBB by reduced efficacy of P-glycoprotein
Epilepsy	Transient BBB opening in epileptogenic foci, and upregulated expression of P-glycoprotein and other drug efflux transporters in astrocytes and endothelium
Brain tumors	Breakdown of the BBB Downregulation of tight junction protein claudin 1, 3, and occludin; redistribution of astrocyte AQP4 and Kir4.1 (inwardly rectifying K+ channel)
Pain	Inflammatory pain alters BBB tight junction protein expression and BBB permeability
Glaucoma	Opening of the BBB, possibly through the diffusion of endothelin-1 and matrix-metalloproteinase-9 into peri-capillary tissue
Lysosomal storage diseases (LSD)	May show changes in BBB permeability, and/or transport, depending on specific LSD

### Ischemic stroke

There are extensive studies showed the effect of hypoxia–ischemia on the BBB, which suggested the disruption of the TJs and the increase in BBB permeability. These events seem to be mediated by released soluble factors, including cytokines, vascular endothelial growth factor (VEGF), and NO. Elevated levels of proinflammatory cytokines, IL-1β, and TNF-α have been reported in animal brains after focal and global ischemia [174] and in CSF of stroke patients [175]. An in vitro study on the BBB model consisting of human cerebrovascular endothelial cells and astrocytes reported that simulated ischemia induces IL-8 and monocyte chemoattractant protein-1 (MCP-1) secretion from endothelial cells and astrocytes [176]. A different study by the same group of investigators observed that human astrocytes under in vitro hypoxic conditions release inflammatory mediators that are capable of up-regulating genes of IL-8, ICAM-1, E-selectin, IL-1 β, TNF-α, and MCP-1 in human cerebrovascular endothelial cells [177]. High levels of cytokines result in the up-regulation of endothelial and neutrophil adhesion molecules. This phenomenon subsequently leads to the transmigration of leukocytes across the endothelium and the BBB. The reported increase in phosphotyrosine staining, loss of TJs molecules (e.g., occludin and ZO), along with the apparent redistribution of AJs protein (such as vinculin), suggest that leukocyte recruitment may trigger signal transduction cascades that result in disorganization of TJs and BBB breakdown [178].

Another study using ^14^C sucrose, a marker of paracellular permeability, showed a 2.6-fold increase in sucrose permeability upon exposing primary bovine brain microvessel endothelial cells to hypoxic conditions. They also reported an increased expression of actin, and changes in occludin, ZO-1, and ZO-2 protein distribution [179]. In summary, these investigations suggest that TJs disruption and increased BBB permeability triggered by hypoxia–ischemia involves a cascade of events in which cytokines, VEGF, and NO are the leading players and astrocytes appear to play a protective role. Further in vivo studies are needed to validate these results as most of these experiments are performed on in vitro models.

### Brain tumors

An increased vascular permeability has been reported in brain tumors due to the poorly developed and compromised BBB [180]. Studies have shown that there is a disruption of interendothelial TJs in human gliomas and metastatic adenocarcinoma [181]. The claudin-1 expression is lost in the microvessels of glioblastoma multiform, whereas claudin-5 and occludin are significantly down-regulated, and ZO-1 expression is unaffected [62]. On the other hand, the reported opening of endothelial TJs in astrocytoma and metastatic adenocarcinoma may be attributed to the loss of the 55 kDa occludin expression in the brain microvessels [78].

There is no clear explanation for the loss of TJs molecules in brain tumor microvessels. However, VEGF and cytokines [182], secreted by astrocytoma and other brain tumors, maybe the key players involved in down-regulating TJs molecules, increased vascular permeability, and cerebral edema. Neoplastic astrocytes are poorly differentiated, which may not be able to release factors necessary for BBB function.

Water channel molecule, aquaporin-4 (AQP4), has been suggested to play a role in BBB disruption in tumors as cerebral edema is an important sign of brain tumor. Multiple investigations have shown that AQP4 is extensively up-regulated in astrocytoma and metastatic adenocarcinoma, which correlates with the observed BBB opening visualized by contrast-enhanced computed tomograms [183]. Animal studies also highlight the AQP4 role in brain edema as Mice deficient in AQP4 have much better survival than wild-type mice in a model of brain edema caused by acute water intoxication. Besides the reported up-regulation of AQP4 in rat models of ischemia [184] and brain injury [185]. Thus, it seems that BBB disruption associated with brain tumors and other forms of brain insults up-regulate the expression of AQP4. Still, the exact mechanism behind the increased expression of AQP4 in different clinical situations is not known.

30 percent of tumors in the brain are metastatic lesions produced by cancers such as lung, breast, and melanoma [186]. The phenomenon surprisingly happens even though the brain is highly impermeable to cancerous cells and prevents their entry into the CNS. Partial disruption of the BBB could be an explanation for this and colonization of the tumor cells in the brain. Besides, this can be explained by transendothelial migration of tumor cells which principally resembles transendothelial migration of leukocytes i.e. rolling, adhesion, and diapedesis.

Certain chemotherapeutic agents can inhibit the growth of tumor cells outside the CNS while they are incapable of affecting the cells inside the brain as an example, trastuzumab in HER2-positive breast cancer [187]. This can be explained by the limited permeability of the agents through the BBB, which leads to sub-therapeutic concentrations in the brain [188]. Lockman et al. [189] showed that the BBB remains partially intact in experimental brain metastases and thus impair drug delivery, requiring a need for brain permeable molecular therapeutics. Similarly, Osswald et al. [190] showed that only the brain permeable compounds inhibit the growth of impermeable lesions in the brain compared to brain impermeable compounds.

### Septic encephalopathy

The CNS pathophysiology in septic encephalopathy represented in decreased cerebral blood flow and oxygen extraction by the brain cells, cerebral edema, and BBB disruption may be related to several reasons including the effect of inflammatory mediators on the cerebrovascular microvessels, abnormal neurotransmitter composition of the reticular activating system, impaired astrocyte function, and neuronal degeneration [191]. Permeability studies in rodents using colloidal iron oxide [192], ^14^C amino acid [193], and ^125^I-albumin [194], showed their ability to enter the brain parenchyma under septic encephalopathy condition which suggests the breakdown of the BBB. Also, elevated CSF protein content has been observed. Several cellular pathologies underlying this BBB disruption have been reported in animal studies, including increased pinocytosis in the brain microvessel endothelium and swelling of astrocytes end-feet, detachment from microvessel walls, and dark, shrunken neurons [191, 192]. Studies also suggested the implication of the adrenergic system in the inflammatory response to sepsis where β_2_ adrenoreceptor stimulation seems to be suppressed, and α_1_ adrenoreceptor stimulation appears to trigger an inflammatory response and hence influence BBB permeability [195].

### HIV encephalitis

CNS infection with human immunodeficiency virus (HIV) is associated with immune activation of astrocytes and macrophages. Activated macrophages and astrocytes release cytokines, chemokines, reactive oxygen species, and several neurotoxins, which impair cellular functioning, alter transmitter action, and result in leukoencephalopathy and neuronal dysfunction [196]. The severe neurologic pathologies seem to be attributed to TNF-α along with other neurotoxins such as arachidonic acid, NO, platelet-activating factor, and quinolinic acid. HIV-infected macrophages are released mainly TNF-α, which particularly affects oligodendrocytes [197]. It is not fully understood how the virus enters the CNS, but once there, it compromised BBB integrity, which facilitates viral entry to the brain and exaggerates the neuronal injury. Studies have shown serum protein leakage in the brains of HIV-associated dementia patients besides accumulation in subcortical neurons and glia [198]. Structural proteins of TJs, such as ZO-1 and occludin were absent or fragmented in brains of patients died from HIV-1 encephalitis where such changes was not observed in patients without encephalitis [199].

The gp120, an envelope glycoprotein, expression in HIV-1 gp120 transgenic mice cause extravasation of albumin and induce the expression of cellular and vascular adhesion molecules such as ICAM-1 and VCAM-1. Also, circulating gp120 has been shown to affect BBB integrity in the transgenic mice [200, 201]. Different studies reported that gp120 is cytotoxic to HUVECs and other ECs from the brain and lungs, which may be attributed to different factors including induced gelatinolytic activity, higher expression of metalloproteinases, and/or induced oxidative stress [202, 203].

### Alzheimer’s disease (AD)

Amyloid beta (Aβ), a 36–43 amino acid peptides, is one of the main constituents of the amyloid plaques found in the brain of people with Alzheimer’s disease. The high levels of accumulated β-amyloid protein and related oligopeptides in the brain of diseased people activate microglia and astrocytes, which lead to a cascade of events producing toxic molecules, neuronal damage, and synaptic dysfunction [204]. Macrophages or microglia, associated with β-amyloid plaque, get activated and interact with astrocyte resulting in the release of different cytokines including interleukin-1 β, TNF-α, transforming growth factor-β, neurotrophic factors such as NGF and bNGF, and reactive oxygen species [205]. Besides, studies showed that β-amyloid stimulates NF-κB that induces the transcription of TNF-α, IL-1, IL-6, monocyte chemoattractant protein-1, and nitric oxide synthetase [206, 207]. Immune cells activation and migration, and the released cytokines affect the BBB integrity.

Amyloid-β is reversely transported from the brain to the blood by binding to LRP1 at the abluminal surface of BBB, which results in its rapid internalization and clearance from the brain. Phosphatidylinositol binding clathrin assembly protein (PICALM) is an essential factor in the internalization and transcytosis of the Aβ-LRP1 complex [208]. Different studies have shown that any mutation to the PICALM gene resulting in decreased expression in ECs may affect disease progression and is considered as a genetic risk factor for Alzheimer’s disease [209]. Furthermore, apolipoprotein E (APOE4), a protein responsible for Aβ clearance, the mutation is considered as the most substantial genetic risk factor for the late onset of AD. Unlike other forms, APOE4 showed to cause BBB disruption and increase fibrinogen and iron in the brain of AD patients. Studies in APOE4 transgenic mice suggested that vascular changes may occur early and proceeded by the neuronal behavioral changes [210].

### Multiple sclerosis (MS)

Multiple sclerosis is an autoimmune disease in which reactive T cells interact with the antigen presented by macrophages- or microglia-expressing HLA-DR2a and HLADR2b, which lead to destroying myelin sheath and the underlying axons [211]. Nitric oxide and cytokines, including interferon-γ, TNF-α, and IL-3, are released by activated macrophages, which damage oligodendrocytes, thus causing interference with myelination and myelin gene expression [212, 213]. Disruption of the BBB is one of the initial critical steps in multiple sclerosis, which follows massive infiltration of T cells and the formation of demyelinated foci. Also, higher levels of reactive oxygen species have been observed in MS lesions, which result in brain damage and contribute to several mechanisms underlying the pathogenesis of MS lesions [214]. Lipid peroxidation products and nitric oxide metabolites are reported to be elevated in the serum of patients with MS [215].

## Markers to assess the blood–brain barrier integrity in vitro or in vivo

The raised interest in exploring BBB—after many studies showed its critical role in a wide range of neurological disorders—mandate the use of accurate and representative markers to demonstrate the integrity of the barriers between the blood, the brain, and the CSF [216]. The drug used should be metabolically inert, non-toxic at the applied doses, not bound to other molecules such as proteins in plasma or tissues, be available in a range of molecular sizes, can be visualized in the range from the naked eye to the electron microscopical level, and be reliably quantifiable.

To date, there is no single available marker that fulfills all the criteria mentioned above. Dextran, available in a wide range of molecular sizes labeled with either biotin or a fluorescent tag, has been extensively used in permeability studies, but it is tedious to quantify. Radiolabeled markers, which are also available in a wide range of molecular sizes, can be easily quantified. Still, they cannot be visualized with enough resolution and have many other limitations, which will be mentioned below. Recently ^13^C sucrose has been introduced as a small molecular weight marker, which can be precisely quantified but also cannot be visualized.

For the quantification of subtle BBB impairment, markers with small molecular weight, such as sucrose should be used, because even a minor degree of barrier damage is expected to have a noticeable effect on their permeability which cannot be quantified with larger molecular weight markers [217]. In general, physicochemical properties, such as size and polarity should be considered for measuring the BBB permeability. When the BBB is compromised and loses its integrity, the chance of large molecular weight markers such as dextran to enter the brain increases, and this could be considered as an option to evaluate the integrity and permeability of the BBB however, for accurately examining small changes in BBB permeability that occur in many neurological conditions, small molecular weight markers (with MW of less than 400 Da) have superiority. Thus, a combination of different markers is currently the most reliable approach to sufficiently assess barrier integrity in the developing or pathological brain. We will try to briefly explore the most used markers and elaborate on their advantages and disadvantages, hoping that will help researchers pick the best marker which can fit their application. A summary of different markers used for BBB permeability studies is represented in Table 2.

**Table 2:** Summary of different markers that can be used for BBB permeability studies

Marker	Size (Da)	Binding		Advantage	Disadvantage
		Protein	Tissue		
Radiolabeled-mannitol	182	No	No	No interaction with proteins Metabolically stable Uncharged No interaction with BBB transporters Suitable for small molecules permeability prediction	Contains lipophilic impurities Requires a radioactive license High cost It cannot be visualized
Biotin ethylenediamine	286	No	No	It can be measured quantitatively with HPLC Visual qualification is feasible	It has a low binding to plasma proteins
Radiolabeled-sucrose	342	No	No	No interaction with proteins Metabolically stable Uncharged No interactions with BBB transporters Suitable for small molecules permeability prediction	Contains lipophilic impurities Over-time degradation Requires a radioactive license High cost It cannot be visualized
^13^C_12_ sucrose	354	No	No	Non-radioactive A sensitive and specific method of detection No interaction with BBB transporters Metabolically stable No interaction with protein Suitable for small molecules permeability prediction	Requires LC–MS/MS device for detection High cost It cannot be visualized
Sodium fluorescein	376	Weak	NR	Easy detection method Freely diffusible Detectable in very low concentrations Inexpensive Non-radioactive Nontoxic It can be visually assessed Suitable for small molecules permeability prediction	Interaction with BBB transporters Weekly binds to plasma proteins
Evans blue	961	Yes	Yes	Visual investigation and qualification are feasible using different microscope techniques Allow assessment of vascular permeability to macromolecules due to binding to its extensive binding to albumin	Quantification is unreliable It strongly binds to serum albumin in vivo and in vitro and, thereby, becomes a high molecular weight protein tracer (69 kDa) Potential in vivo toxicity Not suitable for small molecules permeability prediction
Trypan blue	961	Yes	Yes	Visual evaluation is feasible	Quantification is unreliable Binds to plasma proteins Not suitable for small molecules permeability prediction
Radio-inulin	7000	No	No	Quantification is feasible No protein binding	Contains lipophilic impurities Requires a radioactive license High cost It cannot be visualized
Horseradish peroxidase	44,000	NR	NR	Can be easily visualized under light and electron microscopy	The possibility of diffusion artifacts resulted in a distribution of the reaction product that may not reflect actual protein Found to be toxic in large doses Not suitable for small molecules permeability prediction Quantification is unreliable Species-specific degranulation of mast cells and histamine release
Albumin	69,000	No	No	It is widely used in the radiolabeled or fluorescently labeled form The fluorescent-labeled version could be used for morphological studies The radiolabeled version allows for accurate quantification	Requires radioactive license Not suitable for small molecules permeability prediction
Dextrans	1500 to 70,000	No	No	It can be used for a broad range of molecular weights Can be visualized with both light microscopic and electron microscopic level due to conjugation with biotin of FITC	Not suitable for small molecules permeability prediction Stability issue May be toxic at high concentrations

*NR* not reported

### Evans blue

Evans blue (T-1824) dye is one of the oldest dyes used in animal and human studies. It was used for a long time for plasma volume measurements. The first use in the assessment of BBB was reported in 1966 by Rössner and Temple [218]. Nowadays, Evans blue is widely used as a high-molecular-weight permeability marker to study capillary and cellular membrane permeability. Evans blue extensively binds to serum albumin as soon as it gets to the vascular system [219]. In the case of BBB disruption, the dye leaks through and stains the brain lesion where the BBB is impaired. The advantage of using Evans blue tracer is that it is easy to visualize the zone of altered permeability [220]. That is very useful in animal studies in which lesions with BBB breakdown in specific brain regions are investigated. For many years, the Evans blue method has been used to assess vascular protein leakage macroscopically [221].

With the advanced detection techniques and the wide availability of other accurate markers, researchers start to favor the use of other markers than Evans blue. Studies over the long period of Evans blue application, unveil many drawbacks which limit its use. These limitations include (1) a substantial amount of free dye being present in an animal following the amounts injected, (2) extensive binding to albumin and species-specific binding to other plasma proteins (researcher mainly used Evans blue to estimate albumin penetration through disrupted BBB), (3) unstable in saline and other salt solution, (4) different studies showed it binds to tissues, (5) inaccurate quantitative assessment of BBB damage due to spectroscopic limitations, Evans blue show spectral shifts in protein-containing solutions, (6) in vivo potential lethal toxicity [216].

### Horseradish peroxidase

Horseradish peroxidase (HRP) has been used for years as a vascular permeability in morphological studies [222]. It is available commercially in several types such as types II, IV, and VI). The importance of the introduction of HRP is that the reaction product of this peroxidase forms electron-dense that can be visualized under electron microscopy. The use of horseradish peroxidase helps to expose the nature and location of the BBB and the critical contribution of the brain endothelium to its formation [223]. The main barrier for this tracer to enter the brain lies within the intercellular tight junction between adjacent endothelial cells and the scarcity of pinocytic vesicles in cerebral microvessels ECs. Also, the tight junctions between the epithelial cells of the choroid plexus showed to restrict the movement of HRP across the blood–CSF interface. Under several experimental and pathological conditions, HRP can cross the BBB, which provides morphological evidence for a barrier opening [224, 225].

There are a few limitations that need to be addressed when using this tracer. HRP can cause degranulation of mast cells leading to the release of histamine and serotonin, which subsequently affect vascular permeability [226]. This phenomenon seems to be species-specific as it was observed in certain strains of rats but not in the others [227]. The problem can be avoided by (1) using different strains such as Wistar rats, which did not show mast cell degranulation upon HRP injection [228]. Another solution is to concurrently treat the animal with antihistaminic and anti-serotonergic agents that will mask the degranulation effect. (2) Altogether, caution must be taken while interpreting the results of experiments using HRP, mainly when large doses were applied, and there was no pretreatment with antihistamines.

### Sodium fluorescein

Sodium fluorescein, a 376 Da molecule, was the first visualizable small molecular-sized marker to be introduced into the BBB field [229, 230]. The required dose for injection into mice for barrier permeability experiments (50 mg/kg body weight) is very low considered to its LD 50 in mice, which was estimated as 4738 ± 1.23 mg/kg body weight [230]. Also, an injection of a single dose of 500 mg/kg in pregnant mice did not show to have any embryotoxic or teratogenic effects [231]. It was suggested that sodium fluorescein could be assayed spectrophotofluorometrically (excitation at 440 nm and emission at 525 nm), which will facilitate its detection in BBB permeability studies [220]. Thus, sodium fluorescein seems to be considerably less toxic than Evans blue or HRP. Unlike the Evans blue dye, it shows only weak binding to plasma proteins, which favor its use as a small molecular size marker for blood–brain barrier integrity.

### Sucrose

The use of radiolabeled [^14^C] sucrose for BBB permeability studies was introduced by Dixon Woodbury, Hugh Davson, and Bill Oldendorf, three famous scientists in the BBB field [232–234]. The importance of using this marker is that it allows a quantitative determination of blood–brain or blood–CSF permeability. Experiments applying sucrose need to be designed carefully by ensuring that steady-state plasma levels of the maker are achieved, blood contamination of brain samples by the marker is estimated and secured isotopic labeling of the marker.

Recently, the disaccharide sucrose is considered the most widely accepted standard for the precise measurement of paracellular BBB permeability [217, 235, 236]. Sucrose is substantially better than other markers as being uncharged, not subjected to protein binding, metabolically stable after parenteral administration, falling within the molecular weight range of most small molecule drugs, and not a substrate for active or facilitative transporters in vertebrates animals [237]. However, radiotracer use is associated with special handling and licensing requirements. Impurities in the dosing solution might significantly impact the outcome of experiments [238, 239]. To overcome the drawbacks of radiolabeled tracer and avoid the non-specificity of total radioactivity measurement, [^13^C_12_] sucrose has been introduced as a superior non-radioactive marker, which can be precisely quantified by a sensitive and highly specific LC–MS/MS technique [240].

For an accurate estimation of the marker in the brain tissue, the method relies on transcardiac perfusion with buffer solution before tissue sampling to remove the marker from the brain vasculature. However, it is difficult to judge the full vascular washout in an individual animal. Besides, there are multiple variations in technical details on how the perfusions are performed, such as concerning total volume, duration, flow rate, temperature, and composition of a perfusion fluid, which may add to the experimental variability [241]. Furthermore, the accuracy of this method is questionable, particularly in experiments involving brain trauma, where part of the cerebral circulation is obstructed by post-trauma blood coagulation within vessels [216].

As an alternative, a second marker can be injected just before the terminal sampling time. This marker must be present in the circulation long enough to mix appropriately but not to penetrate the brain to any measurable extent. A second stable isotope-labeled sucrose variant [^13^C^6^] sucrose, which contains 6 of the carbons in the fructose moiety labeled with ^13^C isotope, can serve as a vascular marker. The method allows the simultaneous measurement of both analytes in the same sample in a single run [241]. For radiolabeled tracers, the use of radiolabeled markers such as ^113m^Indium has been reported [242]. Indium binds to transferrin and has the advantage of a very short half-life, but other radiolabeled markers such as albumin or inulin would also be suitable. Poduslo’s laboratory corrected the brain uptake of macromolecules by radioiodinated the same protein with either ^125^I or ^131^I, and one labeled species was used as a vascular marker [243].

Providing these factors are taken into consideration, the use of sucrose is a valuable way of obtaining an accurate quantitative estimate of any brain barrier dysfunction, particularly with its molecular weight falling within the molecular weight range of most small-molecule drugs. Their disadvantage is that it cannot be visualized in tissue sections, so the morphological nature of the disruption cannot be ascertained.

### Dextrans

Dextrans are complex, branched polysaccharides consisting of many glucose molecules. They are commercially available labeled either with a fluorophore or biotin with their chain length varying from 3 to 2000 kDa. Ethylenediamine, a 286 Da biotin-labeled molecule, is also available, which is smaller than sucrose (342 Da), which is a commonly used marker for paracellular permeability in BBB studies [216].

The currently available biotin and fluorophore-labeled dextrans are highly purified, and only small amounts are required because of the sensitivity of the techniques utilized to visualize them. Biotin-labeled molecules can be visualized both under light and electron microscopy. Its permeability across the blood–CSF barrier is comparable to the permeability of more traditional permeability radiolabeled markers, sucrose, and inulin [244]. The use of fluorophore and biotin-labeled dextrans help in clarifying that the route of entry from the blood to the CSF is an intracellular path via the plexus epithelial cells, [245], rather than intercellularly via the tight junctions as generally believed [2]. In general, labeled dextrans are valuable markers of BBB integrity, which can be used safely in small concentrations. The biotin-labeled form is particularly valuable as it can be visualized under both light and electron microscopy.

### Peripheral markers

Brain-derived proteins may work as markers of BBB integrity as they have several possible mechanisms across the BBB. Under physiologic conditions, the production of CSF from plasma involves an efficient filtration process in the choroid plexus that results in minimal quantities of proteins in the CSF [246]. However, many neurological disorders are associated with elevated CSF protein levels. Proteins in CSF can be detected by directly sampling the CSF (lumbar puncture) or intraoperative sampling from ventricles or the subarachnoid space. BBB integrity can also be assessed by contrast-enhanced computed tomography or MRI [247].

Accurate non-invasive techniques would be preferable, mainly to analyze multiple longitudinal samples. A few species of proteins are found exclusively or almost exclusively in the cerebrospinal fluid. Any dysfunction in BBB may allow protein leakage in both directions. Thus, measuring serum levels of CSF proteins represents a non-invasive approach for evaluating BBB integrity and may be of diagnostic value [248].

Currently, only invasive and expensive techniques such as contrast-enhanced magnetic resonance imaging, CT-scan, and lumbar puncture are available to assess BBB integrity clinically. Detection of alterations in blood composition has been proposed as an alternative way to predict BBB disruption [248]. Distinguishing between BBB defects and neuronal damage has immense clinical significance. In ischemic stroke, the delay between insult and the irreversible neuronal cell death offers a window of therapeutic opportunity. If BBB openings develop early after the initial arterial occlusion [249, 250], clinicians would have a unique opportunity to administer drugs that are usually BBB impermeant (e.g., nerve growth factors) before neurons were damaged. The opening time may be unpredictable, so a peripheral, non-invasive, easily repeatable test would be instrumental.

**Fig. 4:**
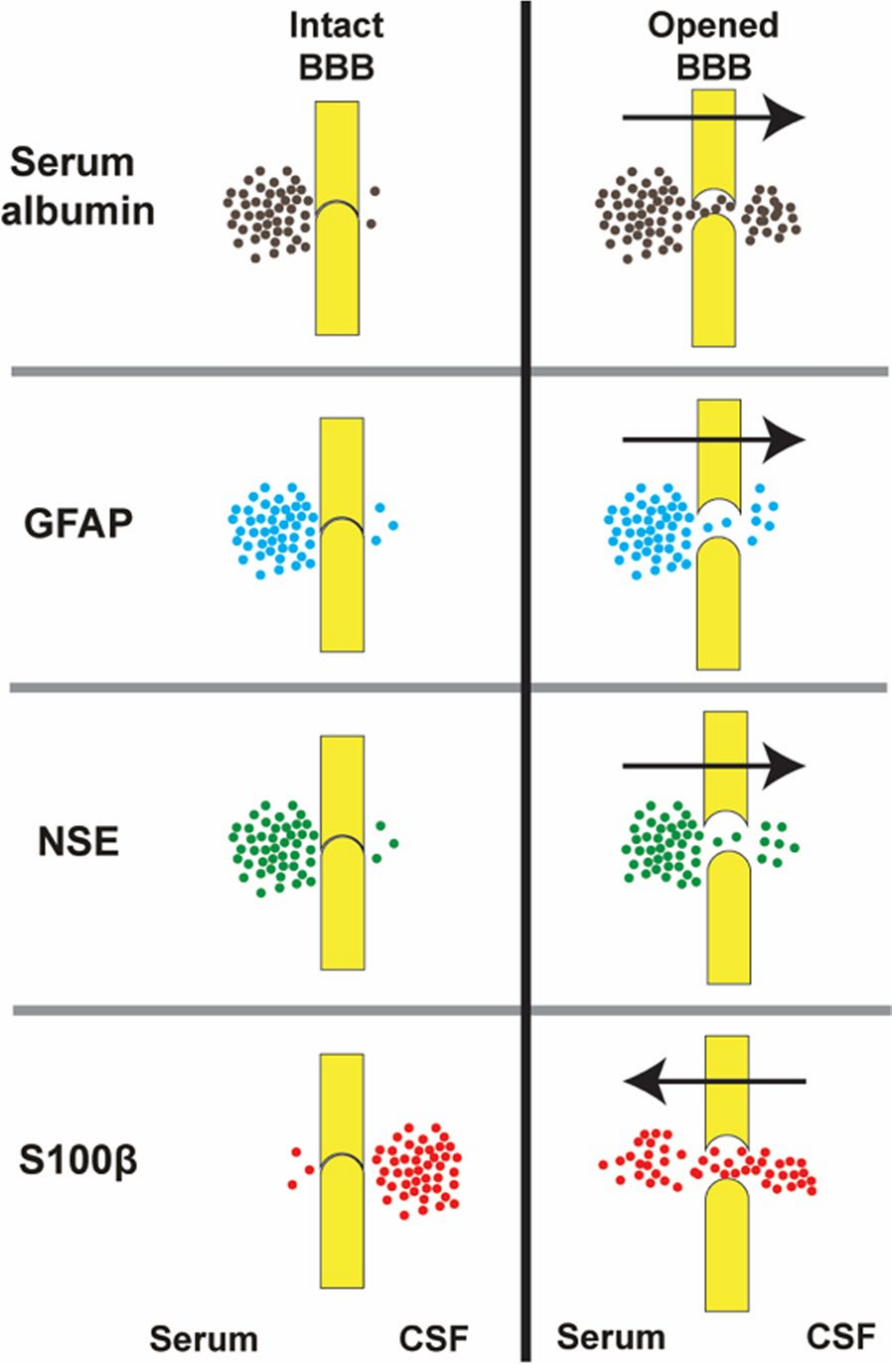
Protein biomarkers protein and their side selectivity. *GFAP* glial fibrillary acidic protein, *NSE* neuron specific enolase

Because of the high interest in neuronal damage, many of the previous studies on biochemical markers have focused on targets that measure neuronal damage. However, most neurologic diseases are associated with increased BBB permeability, and thus the markers thought to indicate neuronal damage may indicate BBB dysfunction. Marker proteins under investigation have included neuron-specific enolase (NSE), glial fibrillary acidic protein (GFAP), and S100β (see Fig. 4) [251].

S100β seems to be promising as its level is more correlated with BBB integrity rather than with neuronal damage, unlike monomeric transthyretin (TTR), a neuronal protein, that may be considered a potential marker of opening to the blood–CSF barrier [252]. In normal subjects, NSE is more concentrated in plasma, and S100β is primarily present in central nervous system fluids. Thus, opening the blood–brain barrier in the absence of neuronal damage would be expected to increase plasma S100β levels while leaving NSE levels markedly unchanged.

S100β is primarily synthesized in the brain by the end feet process of the astrocytes where it accumulates. When the BBB is disrupted, S100β is quickly released in the blood circulation [253, 254]. S100β has also been found in other tissues but at lower concentrations [255, 256]. While S100β appearance in plasma correlated well with BBB openings, S100β has been shown to increase in plasma, the CSF, or both as a consequence of other pathologies not limited to the brain. According to these authors, low levels of S100β are ordinarily present at the blood-to-brain interface and in the CSF. At the same time, disruption of the BBB will result in the sudden appearance of cerebral S100β in serum [257].

## Conclusions

The BBB is a fundamental component of the CNS. Its functional and structural integrity is vital in maintaining the homeostasis of the brain microenvironment. Deterioration in BBB function may play a significant role in the pathogenesis of disease since the BBB dynamically responds to many events associated with flow disturbances, free radical release, and cytokine generation. Furthermore, many neurological disorders and lesions are associated with increased BBB permeability such as neoplasia, hypertension, dementia, epilepsy, infection, multiple sclerosis, and trauma. Any disorder which affects BBB function will cause secondary effects on cerebral blood flow and vascular tone, further influencing transport across the BBB. In this review, we covered several critical aspects of the BBB encompassing the role and functions of its cellular components as well as the contribution of physical stimuli such as shear stress. We also examined several neurological disorders related to the impairment of the BBB. Finally, we provided a comprehensive list of methods currently available to assess the viability of the BBB in vivo and in vitro and discuss the pros and cons of each method.

## Data Availability

Not applicable.

## Abbreviations

BBB: Blood–brain barrier
CNS: Central nervous system
CSF: Cerebrospinal fluid
BCB: Blood–CSF barrier
ISF: Interstitial fluid
TJs: Tight junctions
P-gp: P-glycoprotein
BCRP: Breast cancer resistance protein
OATP: Organic anion transporting polypeptide
CYP 450: Cytochrome P450
BM: Basement membrane
PCs: Pericytes
NVU: Neurovascular unit
ECs: Endothelial cells
PDGFR-b: Platelet-derived growth factor b-receptor
RGS5: Regulator of G-protein signalling-5 (RGS5)
Ajs: Adherens junctions
JAMs: Junction adhesion molecules
ZO: Zonula occludens
EAE: Encephalomyelitis
GBM: Glioblastoma multiforme
MAGUKs: Membrane-associated guanylate kinase-like protein
GUK: Guanyl kinase-like
ROS: Reactive oxygen species
NO: Nitric oxide
HBMECs: Human brain microvascular endothelial cells
TEER: Trans-endothelial electrical resistance
TM: Thrombomodulin
TNF-α: Tumor necrosis factor-α
Il-6: Interleukin-6
MW: Molecular weight
PSA: Polar surface area
MRP: Multidrug resistance-associated proteins
CMT: Carrier-mediated transport
RMT: Receptor mediated transport
SLC: Solute carrier
AMT: Adsorptive-mediated transcytosis
LDL: Low density lipoprotein
LRP1: Lipoprotein receptor-related protein 1
MAb: Monoclonal antibody
TRF: Transferrin receptors
DHA: Docosahexaenoic acid
MFSD2a: Major facilitator superfamily domain-containing protein 2a
ICAM1: Intercellular adhesion molecule 1
VCAM-1: Vascular cell adhesion molecule 1
LFA-1: Lymphocyte function-associated antigen 1
VLA4: Very late antigen 4
VEGF: Vascular endothelial growth factor
MCP-1: Monocyte chemoattractant protein-1
AQP4: Aquaporin-4
HIV: Human immunodeficiency virus
PICALM: Phosphatidylinositol binding clathrin assembly protein
APOR4: Apolipoprotein E
MS: Multiple sclerosis
HRP: Horseradish peroxidase
NSE: Neuron-specific enolase
GFAP: Glial fibrillary acidic protein
TTR: Monomeric transthyretin
